# Controlling Charge Transport in Molecular Wires through Transannular *π–π* Interaction

**DOI:** 10.3390/ma15217801

**Published:** 2022-11-04

**Authors:** Jianjian Song, Jianglin Zhu, Zhaoyong Wang, Gang Liu

**Affiliations:** 1 School of Petroleum Engineering, Yangtze University, Wuhan 430100, China; 2 Southern Marine Science and Engineering Guangdong Laboratory (Zhanjiang), Zhanjiang 524000, China; 3 Key Laboratory of Drilling and Production Engineering for Oil and Gas, Hubei Province, Wuhan 430100, China; 4 China Oilfield Services Ltd. (Blue Ocean BD Hi-Tech Co., Ltd.), Quanzhou 362800, China

**Keywords:** charge-transfer, molecular electronics, transannular *π–π* interaction, [2.2]paracyclophane-1,9-dienes (PCD)

## Abstract

This paper describes the influence of the transannular π–π interaction in controlling the carrier transport in molecular wires by employing the STM break junction technique. Five pentaphenylene-based molecular wires that contained [2.2]paracyclophane-1,9-dienes (PCD) as the building block were prepared as model compounds. Functional substituents with different electronic properties, ranging from strong acceptors to strong donors, were attached to the top parallel aromatic ring and used as a gate. It was found that the carrier transport features of these molecular wires, such as single-molecule conductance and a charge-tunneling barrier, can be systematically controlled through the transannular π–π interaction.

## 1. Introduction

Single-molecule electronics (SME) is the study of electrical procedures measured or controlled on a molecular scale. Charge-transport property regulation is one of the main challenges in the field of molecular electronics, and molecular-scale tuning is the most effective way to address this. Therefore, controlling the charge transport through single-molecule wires is very important for both the understanding of mechanisms and for practical single-molecule applications [1,2,3,4]. It is well-known that both electronic and geometric changes could result in a considerable effect on electric conductance of symmetric single-molecule wire, and the molecule rectification effect of asymmetric single-molecule wire could be rendered by dipole [5,6,7]. Although there are some publications concerning both experimental measurements [8,9,10] and theoretical calculations [11,12,13,14,15] on charge-transport properties, regulating the transport of the charge in a controllable manner (e.g., de/protonation, photon absorption, isomerization, oxidation/reduction) with single molecules is still a challenging subject [16,17,18,19,20].

Cyclophane chemistry has attracted much interest over the last 5 decades due to its unique geometric structure and electronic properties. It has been widely used in various aspects, such as molecular self-assembly [21,22], molecular recognition [23], and optoelectronic polymers [24]. One of the most widely investigated molecules is [2.2]paracyclophane-1,9-dienes (PCD), which was first reported by Cram in 1958 [25], where the two double-bond bridges between the two aromatic rings of the PCD molecule greatly shorten the transannular ***π–π*** distance and strengthen the intramolecular ***π–π*** interaction of the two aromatic rings, in contrast with the saturated bridges of [2.2]paracyclophanes [26,27]. The strong transannular ***π–π*** interaction also leads to a distorted π-electron system and a highly strained molecular structure, which is of importance as PCD molecules can be ring-opened by ring-opening metathesis polymerization to produce poly(1,4-phenylenevinylenes)-based conjugated polymers for the fabrication of functional devices [24,28,29]. Recently, the through-space charge-transfer property of dioctyloxy diperfluorohexyl-substituted PCD molecules was investigated by Yu et al., which also implies the importance of the transannular ***π–π*** interaction in regulating the electronic property of PCD molecules [30].

**Figure 1:**
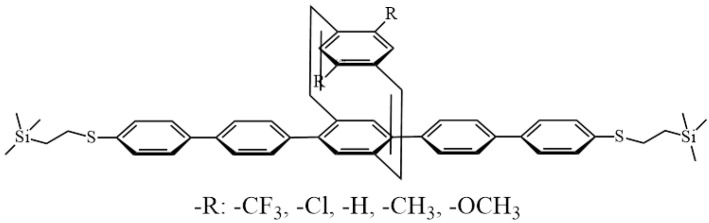
Chemical structure of the PCD molecular wires.

However, aromatic ***π–π*** interaction, which is a noncovalent interaction, has been rarely used as a tuning factor to control the charge-transport process in molecular electronics [31,32,33]. In this paper, we have designed and prepared five single-molecule wires bearing a PCD building motif (see our detailed synthesis of ESI†), and we investigated their molecular electronic properties using the STM-BJ technique. Figure 1 shows the designed structure of these molecular wires, where the top parallel aromatic ring is attached to the conjugated pentaphenylene part by two vinyl groups. Both the *π*-orbital of the top phenyl ring and the vinyl groups are orthogonal to the charge-transport direction along the molecular wire (Figure 1). This structural design allowed us to adjust the electronic interaction between the parallel aromatic ring and the charge-transport channel (pentaphenyl backbone) by adjusting the electronic properties of different functional substituents (R groups) on the top aromatic ring. The top parallel aromatic system can act as a simulated chemical gate to control the conductance of the single-molecule wire, the energy level of the orbit, and the tunneling barrier of the charge from the molecular wires.

## 2. Materials and Methods

### 2.1. Materials

The commercially available chemicals were bought from TCI, except for the tetrakis (triphenylphosphine) palladium, which was purchased from Aldrich. Toluene and tetrahydrofuran (THF) were dried over sodium/benzophenone and freshly distilled prior to use. Other reagents were used without further purification. Compounds 6a, 6b, 6c, 6d, 6e, and 7 (Figure S1, ESI†) were prepared according to the standard methods in the literature [34].

### 2.2. Characterizations

A KSV (Helsinki, Finland) CAM 200 contact angle device was selected to detect the contact angle. An Escalab-250Xi (Thermo-Fisher, Waltham, MA, USA) containing monochromatic Al Kα as a radiation source was used to study the X-ray photoelectron spectroscopy (XPS). ^1^H nuclear magnetic resonance (^1^H NMR) spectra were recorded on a Bruker AV400 spectrometer (Billerica, MA, USA). Matrix-assisted laser desorption/ionization with time-of-flight (MALDI-TOF) mass spectrometry was carried out on a Bruker Ultraflextreme MALDI-TOF/TOF spectrometer using a matrix of dithranol.

### 2.3. Fabrication of Self-Assembled Monolayers (SAMs)

First, a propane flame was used to anneal the chosen substrate, and then the SAMs of the isolated molecules were fabricated according to a previous publication. Briefly, tetrabutylammonium fluoride (TBAF) was used as the deprotecting agent with a tetrahydrofuran (THF) solution containing ca. 1 × 10^−4^ M of the target molecule. After 4 h of immersion in PCD solution, the gold substrate was then washed with THF and ethanol, dried with N_2_, and used immediately.

### 2.4. Single-Molecule Junctions by Break Junction (STM-BJ)

Briefly, a 0.25 mm gold wire was prepared as the gold tip by mechanical cutting. All the experiments were conducted in degassed mesitylene in order to decrease the chance of surface contamination. After that, the STM Teflon solvent holder was sonicated in acetone and dried under N_2_. Then, the solvent holder was placed over the gold–SAM surface, and the designated amount of toluene was added during the measurement. It is estimated that about ~2000 current-distance traces are required in a typical BJ experiment.

### 2.5. I-V Recording of Single-Molecule Junctions

The *I-V* recording experiments were conducted at 100 mV bias voltage in toluene. There are three steps, include tapping, conductance step detection, and *I-V* recording. For the tapping step, when the current increases to a predefined value, the tip will move to the substrate, and then it will retract until reaching a lower, pre-set current. During each retraction, the conductance step detection was applied, and we selected the measured conductance range according to a previously measured conductance histogram. The *I-V* recording step was performed immediately upon the application of the conductance step, and the whole procure included three sub-steps. The first was the immediate holding in position of the tip. The second was the quick (10 Hz) current–voltage curve recording (from +1.5 V to −1.5 V (or +1.8 V to −1.8 V)). The last sub-step occurred when the *I-V* curve detection was completed; the tip would be removed from the substrate once the current decreased to a pre-set value, and the test was started again. For the data selection, *I-V* curves that were incomplete or contained large switching noises were detected and removed from the statistical analysis. This procedure allowed us to obtain complete *I-V* curves for statistical analysis.

Energy offsets *φ*_0_ and the voltage division factor *γ* of the molecular junctions were calculated, following the work by Baldea, with the Equation [35]:

(1) $$\varphi_{0}=eV_{t,a}=\frac{2e\left|V_{t,n}V_{t,p}\right|}{\sqrt{{V_{t,p}}^{2}+10V_{t,p}V_{t,n}/3+V_{t,n}{}^{2}}}$$

(2) $$\gamma =-(\varphi_{0}/V_{t,p}+\varphi_{0}/V_{t,n})/4$$

## 3. Results

### 3.1. Synthesis and Characterization of PCD-Based Molecular Wires

The synthetic routes for the PCD-based molecular wires with different substituents are shown in the Supporting Materials in detail (Figure S1, ESI†). First, [3.3]dithiacyclophanes intermediates (compounds 8a–e) were prepared through the cyclization reaction of benzylic dithiols (compounds 6a-e) and benzylic dithiol (compound 7) in a dilute solution. Then, Stevens rearrangement, oxidation, and pyrolysis treatments led to dibromo-[2.2]paracyclophane-1,9-diene (compounds 9a–e) in a yield of ~15% after flash column chromatography purification. A Suzuki coupling reaction between B-pinacol (Bpin) functionalized biphenyl compounds (compound 5) and dibromo-[2.2]paracyclophane-1,9-diene (compounds 9a–e) furnished the target PCD-based molecular wires (compounds 10a–e). The top parallel phenyl rings were substituted with several groups possessing different electronic demands, which may play a significant role in controlling the transannular π–π interaction between the two parallel aromatic systems to adjust the charge transport of the molecular junction. More specifically, substituents -CF_3_, -Cl, -H, -CH_3_, and -OCH_3_ were immobilized on the top phenyl ring, ranging from a strong electron acceptor to a strong electron donor. For the convenience of discussion, the molecular wires have been named “PCD-X”, where X represents the substituent group. Detailed characterizations of the intermediate products and the final PCD-X molecular wires were carried out by combining ^1^H-NMR (Figures S2–S4 ESI†), MALDI-TOF-MS (Figures S5–S9 ESI†), and UV–Vis spectroscopy (Figure S10 ESI†).

In principle, two isomers of dibromo-[3.3]dithiacyclophanes (compounds 8a–e) intermediates are expected as products during the cyclization process, namely, pseudo-gemini (eclipsed conformation of substituents on the aromatic rings) and pseudo-ortho isomers. However, only one of the possible isomers was recognized on the TLC plate and successfully purified as the main product after column chromatography, with an overall yield of around 40–50%. The clear ^1^H-NMR spectra for all five of the dibromo-[3.3]dithiacyclophanes (Figure S2 ESI†) and the corresponding dibromo-[2.2]para cyclophane-1,9-dienes (Figure S3 ESI†) also supported the finding that only one isomer was obtained.

To unambiguously verify the atom configuration of the prepared isomer, the crystal structures of dichloro-dibromo-[2.2]paracyclophane-1,9-diene (compound 9b) were investigated, and the crystallographic data can be found in ESI†. Single crystals were grown through the slow diffusion of MeOH into the concentrated CH_2_Cl_2_ solutions of both compounds. The X-ray crystal structure (Figure S11 ESI†) of compound 9b clearly confirmed the pseudo-ortho isomer configuration. The selectivity for this specific isomer during the cyclization process appears to be determined by the steric influence between the bulky bromine atoms and the functional groups (CF_3_, Cl, CH_3_, and OCH_3_), which excludes the influence of isomerization on the electronic property of the prepared molecular wires. In addition, two phenyl groups in cyclophane are closely stacked with a short distance of 2.97 Å; therefore, the π electron clouds of the phenyl rings can strongly overlap, and electronic tuning can be performed more effectively. UV–Vis measurement showed that the absorption edges for the PCD-Cl, PCD-H, PCD-CH_3_, and PCD-OCH_3_ molecular wires were around ~350 nm, while a 20 nm red shift was observed for PCD-CF_3_, which is strong evidence that the R group on the top phenyl ring can successfully affect the pentaphenylene backbone via the effective π–π overlap. The trend agrees well with the DFT calculation results (Table S1 ESI†).

### 3.2. Self-Assembled Monolayer Films on Gold

**Figure 2:**
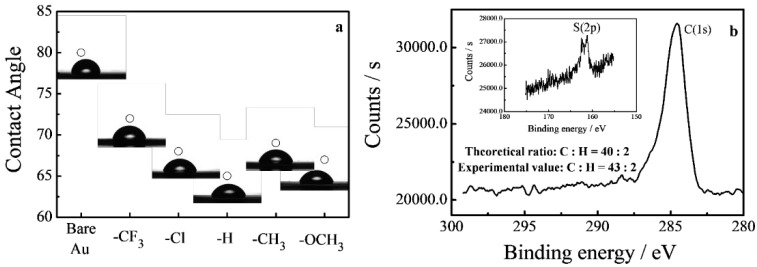
(**a**) Contact angles of the PCD-based self-assembled monolayers, where the statistically average values, based on 3 measurements, were used; (**b**) XPS characterization of the elements C and S of the PCD-H self-assembled monolayer.

Self-assembled monolayer (SAM) films were fabricated on a gold surface, with the reaction of the thiol–gold used to test the conductance of the single molecules, and the molecular junction was based on the reaction of two molecules’ terminal thiol groups with two gold electrodes. Successful immobilization of the molecular wires onto the gold substrates was confirmed by combining contact angle and XPS measurements of the SAMs (Figure 2). Namely, the contact angles (Figure 2a) for the SAMs were found to be 10~15° smaller than that of bare gold, and the similar contact angles observed for all the SAMs indicated that the outermost thiol group dominated the wetting property of the SAM. XPS characterization (Figure 2b) of PCD-H SAM showed that the C/S ratio was 43/2, consistent with the theoretical ratio of 40/2, quantitatively verifying the successful immobilization of PCD molecular wires on the gold surfaces.

**Figure 3:**
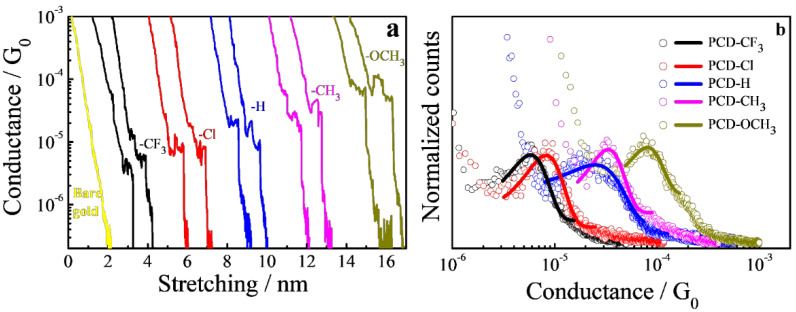
(**a**) Typical conductance–distance traces, and (**b**) conductance histograms of the five PCD molecular wires, where the solid lines represent the Gaussian fitting curves.

The STM-based break junction technique was used to measure the single-molecule conductance [36,37]. With the help of repeatedly forming and breaking the gold point contacts, single-molecule junctions were established at a small bias voltage of 100 mV. The LabVIEW program was utilized to record the conductance traces of the substituted molecules, and Figure 3a exhibits the typical conductance–distance curves of the five PCD molecular junctions. The constant conductance plateaus were between 10^−6^~10^−4^ G_0_ in these stretching traces, which is consistent with the first signature in a single-molecule junction. (G_0_ is the quantum of conductance 2 e^2^/h, e is the electron charge, and h is the Planck constant). The plateau length is commonly around 0.5–1.0 nm, and the conductance fluctuation below 10^−6^ G_0_ may be caused by the noise floor. Figure 3b describes the corresponding conductance histograms calculated from ~400 effective conductance decay curves, which show unambiguous peaks for all of the molecules measured. In addition, the conductance values for the junction were determined by fitting the peak of the Gaussian curve. More specifically, the single-molecule conductance values of the molecular wires PCD-CF_3_, PCD-Cl, PCD-H, PCD-CH_3_, and PCD-OCH_3_ were 5.8 × 10^−6^ G_0_, 8.2 × 10^−6^ G_0_, 2.5 × 10^−5^ G_0_, 3.2 × 10^−5^ G_0_, and 8.1 × 10^−5^ G_0_, respectively. The conductance systematically changed as the substituent varied from strong acceptor (CF_3_) to strong donor (OCH_3_).

Keeping in mind that bias voltage range plays an important role in the charge transport of the single-molecule junction, thousands of current–voltage (*I-V*) curves were recorded for each sample. Thus, the corresponding transition voltage spectroscopy (TVS) results can be obtained. The incomplete and switching *I-V* curves resulting from the breakdown and instability of the molecular junctions were automatically detected but did not appear in the *I-V* histogram. The *I-V* and *G-V* histograms of the five substituted molecules are presented in the supporting information (Figure S12 ESI†). The so-called Fowler–Nordheim (FN) single-molecule TVS plot was obtained by transforming each I–V curve into an ln(I/V^2^) versus 1/V curve [38,39,40,41]. The transition voltage Vt minimum value was compatible with that of the FN plot, and the transition voltage of 1D TVS histograms could be created from these minimum values. 

**Figure 4:**
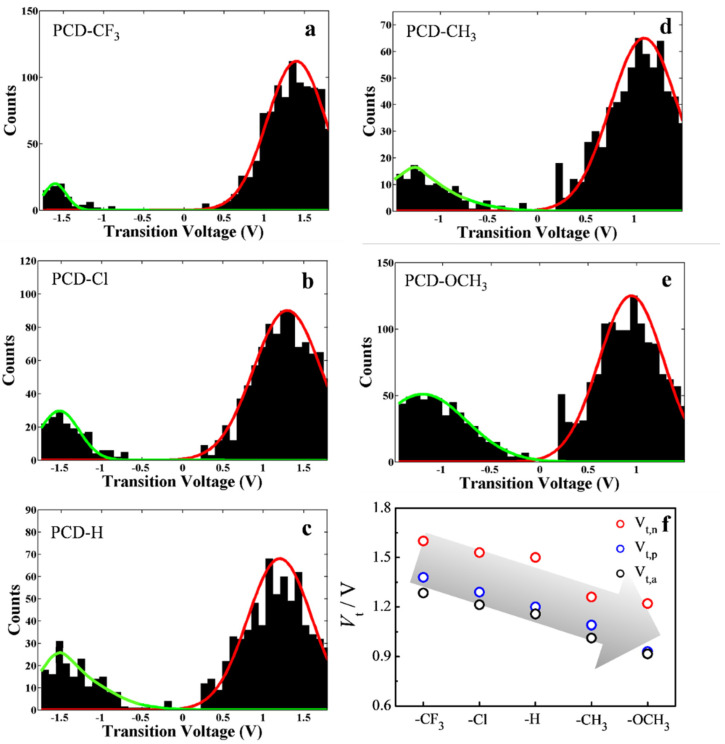
One-dimensional transition voltage histograms of (**a**) PCD-CF_3_, (**b**) PCD-Cl, (**c**) PCD-H, (**d**) PCD-CH_3_, and (**e**) PCD-OCH_3_; (**f**) The negative, positive, and calculated ambipolar transition voltages of the five PCD molecular wires.

Figure 4a–e shows the 1D transition voltage histograms of the five PCD molecules, which present two distinguishing characteristics. One is that all of the histograms are asymmetric, which was previously reported by Kushmerick and Tao [36,37]. The other one is that the absolute values of the experimental positive and negative transition voltages (*V*_t,p_ and *V*_t,n_) decreased from 1.4 V to 0.9 V and from 1.6 V to 1.3 V when the substituent is altered from PCD-CF_3_ to PCD-OCH_3_, and this could be explained as the contact of symmetry molecule and metal-molecule. In addition, the asymmetry could also be caused by the solvent polarity [42].

Considering that the offset energy (φ_0_) between the electrodes’ Fermi level and molecular orbitals are seriously affected by the applied bias V, φ_0_ ≡ φ_0_(V)|V = 0 → φ_0_(V), voltage division factor γ was used to offset the effect, where φ_0_(V) = φ_0_ + γeV [13,30], In particular, the experimentally measured transition voltages V_t,p_ and V_t,n_ for bias polarities could be utilized to evaluate the corresponding ambipolar transition voltage (V_t,a_ = φ_0_/e); the correct energy offset φ_0_ and the voltage division factor γ based on the equations were established by Baldea (Equations (S1) and (S2) ESI†) [41,42]. The calculated Vt,a and γ are summarized in Figure 4f and Figure S13 and in Table S1. As shown in Figure 4f, the V_t,a_ value decreases from 1.3 V to 0.9 V as the substituents change from electron-withdrawing group -CF_3_ to electron-donating group -OCH_3_. In addition, the tunneling barrier heights of the PCD molecules with diverse groups (-CF_3_, -Cl, -H, -CH_3_, and -OCH_3_) were deduced as being around 1.28 eV, 1.21 eV, 1.15 eV, 1.01 eV, and 0.91 eV, respectively. We can thus conclude that the transition voltage and the corresponding charge-tunneling barrier increase with substituent varying from electron donor to electron acceptor. Finally, we calculated the voltage division factor γ, and the values ranged from 0.03~0.06 (Figure S12 ESI†), which confirms a slightly larger potential drop at the soft contact (e.g., the STM tip).

**Figure 5:**
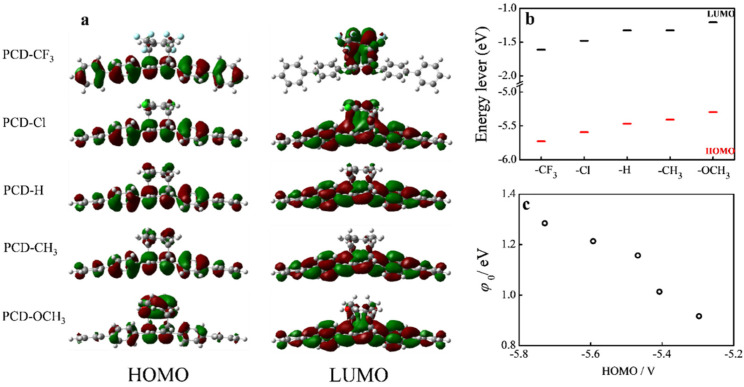
(**a**) Molecular orbital diagrams of PCD molecules, (**b**) Calculated HOMO and LUMO energy levels of these PCD molecules, (**c**) Plot of tunneling barriers (V_t,a_) vs. HOMO energy level, where *φ*_0_ = eV_t,a_.

All of the results discussed above indicate that the top parallel phenyl ring can modulate the molecular conductance and the charge-tunneling barrier of the pentaphenylene conducting channel through the transannular π–π interaction. DFT calculations based on the RB3LYP method, with basis set of 6-31G*, were carried out to obtain insight into the energy-level alignment of these PCD molecular wires. Figure 5a shows that the HOMO starts to shift from the pentaphenylene backbone to the top parallel benzene ring as the substituent varies from electron acceptor -CF_3_ to electron donor -OCH_3_, while the LUMO simultaneously extends to the pentaphenylene backbone. Figure 5b shows that both the HOMO and LUMO energy levels of the molecular wire decrease when the substituent on the top parallel phenyl ring varies from strong donor to strong acceptor. Since the backbone of the molecule is a p-type semiconductor, the hole transport is much easier with the HOMO molecule, which thus benefits the single-molecule conductance of electron-donating groups on the top parallel phenyl [43]. Figure 5c proves how the tunneling barrier φ_0_ changes with the HOMO energy level of the PCD-based molecular wires. It is clear that the φ_0_ decreases as the HOMO energy level increases, indicating that the charge-transport mechanism in these PCD molecular wires is indeed through hole transport and can be regulated by the intramolecular π–π interaction.

## 4. Conclusions

In summary, we have successfully prepared a series of molecular wires that demonstrated how the intramolecular transannular π–π interaction affects the charge-transport property in molecular wires. Both the single-molecule conductance and current–voltage characteristics can be systematically tuned through the transannular π–π interaction. Transition voltage spectroscopy measurement and DFT calculation suggest that the effect is manifested via tuning the HOMO energy level and orbital spatial distribution of these PCD molecules.

## Data Availability

The data can be supported when required.

## Supplementary Materials

The following supporting information can be downloaded at: https://www.mdpi.com/article/10.3390/ma15217801/s1: Figure S1: Synthetic route of the molecular wires; Figure S2: 1H-NMR spectra of compounds **8a**–**8e**; Figure S3: 1H-NMR spectra of compounds **9a**–**9e**; Figure S4: 1H-NMR spectra of compounds **10a**–**10e**; Figure S5: MALDI-TOF-MS spectrum of compound **10a**; Figure S6: MALDI-TOF-MS spectrum of compound **10b**; Figure S7: MALDI-TOF-MS spectrum of compound **10c**; Figure S8: MALDI-TOF-MS spectrum of compound **10d**; Figure S9: MALDI-TOF-MS spectrum of compound **10e**; Figure S10: UV–Vis spectra of the intermediate and final molecular wires in deuterated chloroform; Figure S11: Single-crystal structure of paracyclophane-1,9-dienes; Figure S12: (**a**) Current–voltage 2D histograms, (**b**) conductance–voltage 2D histograms, (**c**) transition voltage 2D histograms, and (**d**) transition voltage 1D histograms of the five molecular wires; Figure S13: Calculated voltage division factor (γ) for the five PCD molecular wires; Table S1: Experimental and DFT calculation results for PCD molecular wires and calculated values of φ0 and γ, based on Equations (1) and (2) as developed by Baldea.
