# Supplementary material for: Controlling Charge Transport in Molecular Wires through Transannular π–π Interaction
Source: Materials (Basel). 2022 Nov 4;15(21):7801. doi: 10.3390/ma15217801 (PMC9654154; doi:10.3390/ma15217801)
Supplement: Supplementary file 1 [file materials-15-07801-s001.zip › materials-1995667-supplementary.pdf]

Controlling Charge Transport in Molecular Wires through Transannular π-π Interaction

Jianjian Song [1,2,3], Jianglin Zhu [2,*], Zhaoyong Wang [4,*] and Gang Liu [4]

1 School of Petroleum Engineering, Yangtze University, Wuhan 430100, China

2 Southern Marine Science and Engineering Guangdong Laboratory (Zhanjiang), Zhanjiang 524000, China

3 Key Laboratory of Drilling and Production Engineering for Oil and Gas, Wuhan 430100, China;

4 China Oilfield Services Ltd. (Blue Ocean BD Hi-Tech Co., Ltd.), Quanzhou 362800, China

* Correspondence: zhujl@zjblab.com (J.Z.); wangzhy16@cosl.com.cn (Z.W.)

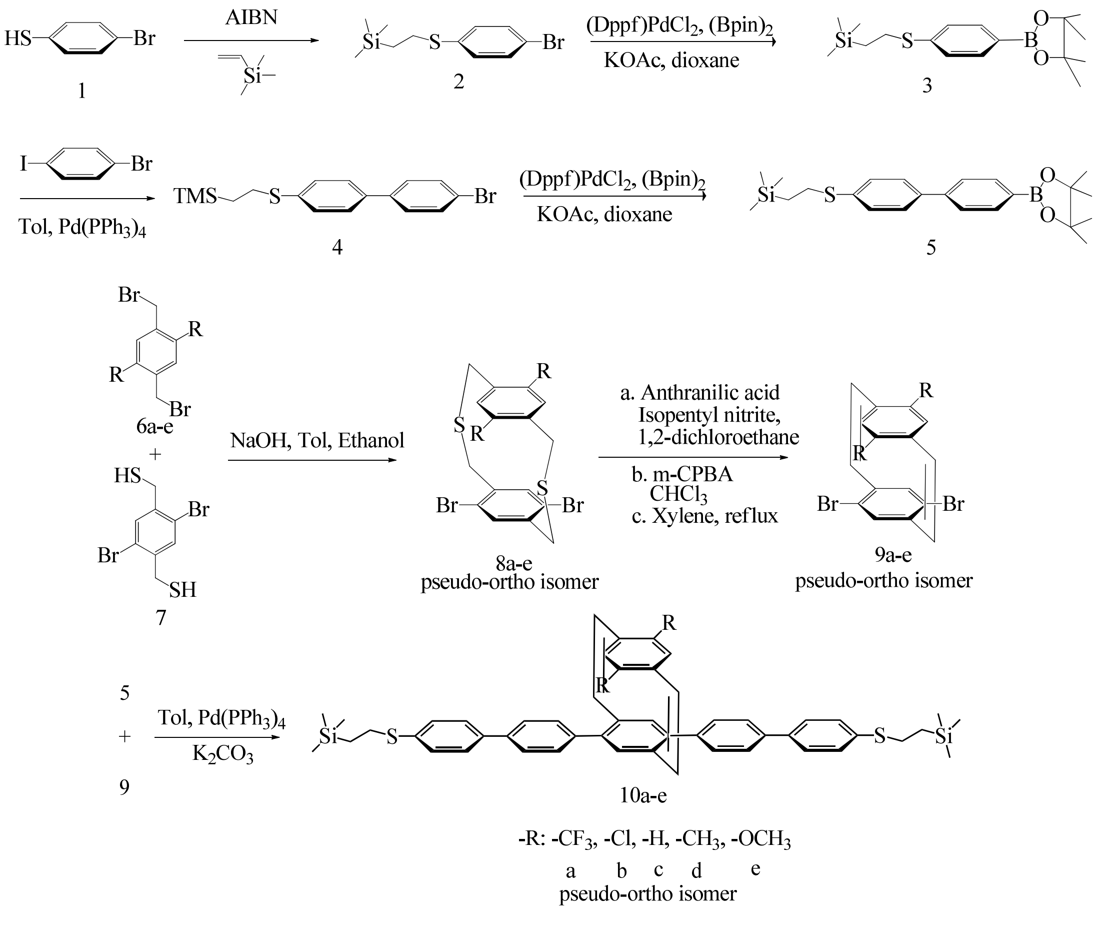

Figure S1. Synthetic route of the molecular wires

Compound 2. To high pressure reaction tube was added **1** (1.00 g, 5.30 mmol), vinyltrimethylsilane (0.921 mL, 6.35 mmol), and azobisisobutyronitrile (9 mg, 0.053 mmol). The tube was sealed and the reaction mixture was stirred and heated at 100 °C for 20 hours. The mixture was then allowed to cool down to room temperature and concentrated under reduced pressure to give **2** as clear liquid (1.35 g, 88% yield). ^{1}H NMR ($CDCl_3$, δ, ppm): 0.04 (s, 9H), 0.89-0.93 (m, 2H), 2.91-2.95 (m 2H), 7.15-7.17 (d, 2H), 7.38-7.40 (d, 2H).

Compound 3. To a solution of **2** (1.35 g, 4.65 mmol) in THF (10 mL) at -78°C under N_2 was added *n*-butyllithium (2.5 M in hexanes) (2.05 mL, 5.12 mmol). After stirring the reaction mixture at -78 °C for 2 hours, trimethyltin chloride (1.0 M in hexanes) (5.12 mL, 5.12 mmol) was added to the mixture. The reaction was allowed to gradually warm to room temperature and stirred for 12 hours. The reaction was diluted with ethyl acetate and water. The organic phase was washed with water, dried with $MgSO_4$ and concentrated under reduced pressure to give **3** as slight yellow oil (1.55 g, 89% yield). ^{1}H NMR ($CDCl_3$, δ, ppm): 0.04 (s, 9H), 0.28 (s, 9H), 0.92-0.96 (m, 2H), 2.94-2.98 (m 2H), 7.27-7.29 (d, 2H), 7.39-7.41 (d, 2H).

Compound 4. To a round-bottom flask equipped with a condenser was added **3** (1.55 g, 4.15 mmol), 1-bromo-4-iodobenzene (1.41 g, 5.01 mmol), and tetrakis(triphenylphosphine)palladium (241 mg, 5 mmol%). The system was evacuated and refilled with N_2 three times, then charged with toluene (50 mL). The reaction mixture was refluxed at 120 °C under N_2 for 16 hours. After cooling to room temperature, the reaction mixture was filtered and concentrated under reduced pressure. Chromatography of the residue over silica gel gave **4** as white solid (712 mg, 47% yield). ^{1}H NMR ($CDCl_3$, δ, ppm): 0.06 (s, 9H), 0.94-0.99 (m, 2H), 2.98-3.02 (m 2H), 7.35-7.37 (d, 2H), 7.43-7.49 (q, 4H), 7.54-7.56 (d, 2H).

Compound 5. To a round-bottom flask equipped with a condenser was added **4** (1.5 g, 4.10 mmol), bis(pinacolato)diboron (1.23 g, 491 mmol), potassium acetate (1.20 g, 12.3 mmol), and [1,1′-Bis(diphenylphosphino)ferrocene]dichloropalladium(II) (200 mg, 6 mmol%). The system was evacuated and refilled with N_2 three times, then charged with dioxane (100 mL). The reaction mixture was bubbled with N_2 for 5 minutes, then refluxed under N_2 for 24 hours. After cooling to room temperature, the reaction mixture was filtered and diluted with dichloromethane. The organic phase was washed with water and brine, dried with $MgSO_4$, and concentrated under reduced pressure. Chromatography of the residue over silica gel gave **10** as white solid (0.93 g, 55% yield). ^{1}H NMR ($CDCl_3$, δ, ppm): 0.06 (s, 9H), 0.94-0.99 (m, 2H), 2.98-3.02 (m 2H), 7.35-7.37 (d, 2H), 7.43-7.49 (q, 4H), 7.54-7.56 (d, 2H).

Compound 8a. To a stirred solution of NaOH (0.526 g, 13.2 mmol) in ethanol (1.8 L) was added dropwise a solution of **6a** (2.4 g, 5.98 mmol) and **7** (1.95 g, 5.98 mmol) in benzene (300 mL) over a period of 8 hours. The reaction was stirred for an additional 10 hours at room temperature. The solution was filtered and concentrated under reduced pressure. Chromatography of the residue over silica gel gave **8a** as white solid. Compounds **8b-8e** were prepared following the general procedure given for the preparation of **8a**. The yields for compounds **8a-8e** were 1.06 g (30%), 1.28 g (38%) , 1.39 g (42%), 0.91 g (29%), and 0.97 g (33%), respectively.

Compound 9a. To a solution of **8a** (1.02 g, 0.170 mmol) in chloroform (50 mL) at -40 °C was added dropwise a solution of m-chloroperoxybenzoic acid (77%) (840 mg, 3.74 mmol) in chloroform (50 mL). The reaction was stirred at -40 °C for 1.5 hours, 0 °C for 30 mins, and room temperature for 2 hours. The solution was then washed with aq. $NaHCO_3$ solution, water, and brine, dried with $MgSO_4$ and concentrated under reduced pressure. The resulting residue was dissolved in

m-xylene (12 mL) and heated to reflux under N_2 for 20 hours. The solution was allowed to cool to room temperature and concentrated under reduced pressure. Chromatography of the residue over silica gel gave **9a** as a white solid. Compounds **9b-9e** were prepared following the general procedure given for the preparation of **9a**. The yields for compounds **9a-9e** were 160 mg (19%), 192 mg (26%), 155 mg (25%), 200 mg (30%), and 144 mg (20%), respectively.

Compounds 10a. To a round-bottom flask equipped with a condenser was added **9a** (105 mg, 0.21 mmol), **5** (188 mg, 0.46 mmol), tetrakis(triphenylphosphine)palladium (20 mg, 8 mmol%), and Aliquat 336 (1 drop). The system was evacuated and refilled with N_2 three times, then charged with toluene (5 mL) and 1 mL of K_2CO_3 (1 M, aq.) The reaction mixture was heated at 85 °C under N_2 for 24 hours. After cooling to room temperature, the reaction mixture was filtered and concentrated under reduced pressure. Chromatography of the residue over silica gel gave **10a** as white solid. Compounds **10b-10e** were prepared following the general procedure given for the preparation of **10a**. The yields for compounds **10a-10e** were 47 mg (25%), 35 mg (20%), 45 mg (29%), 31 mg (19%), and 40 mg (23%), respectively.

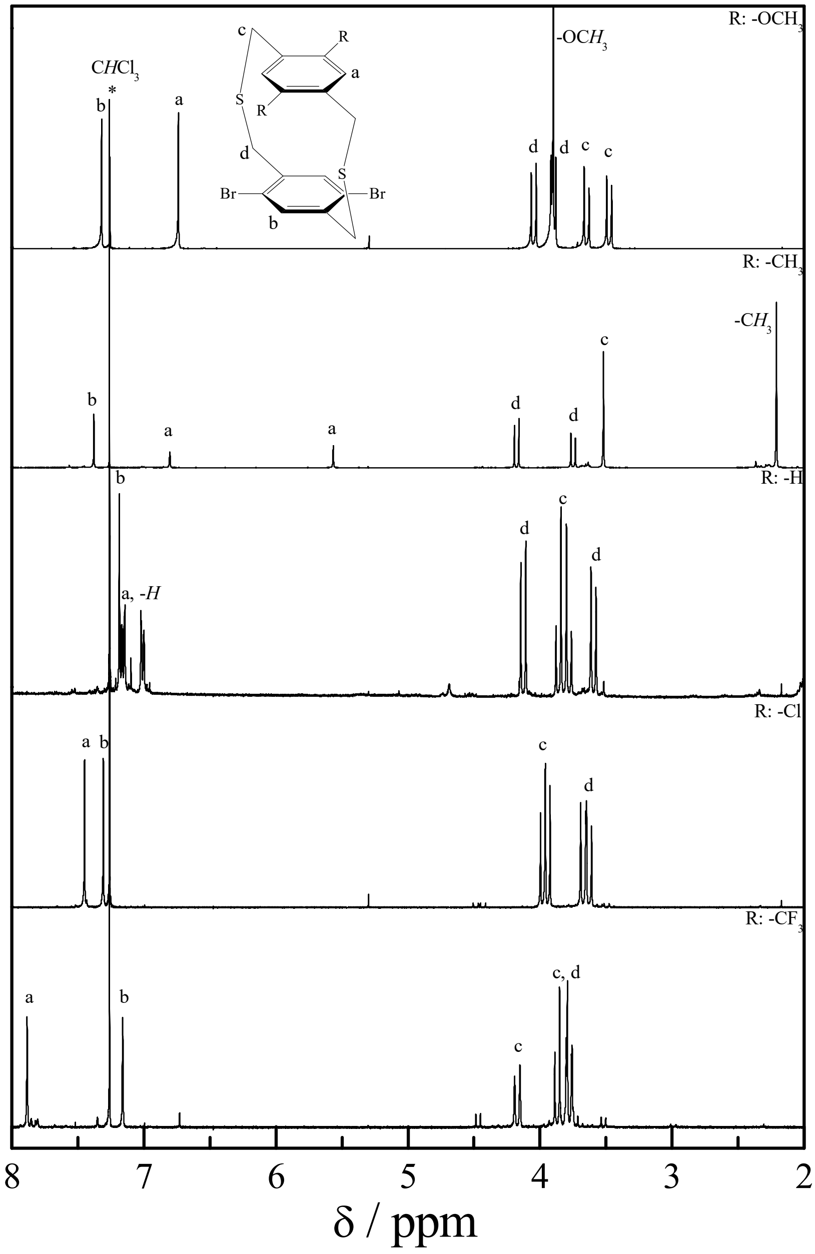

Figure S2. ^{1}H-NMR spectra of compounds 8a-8e (* means the utilized solvent).

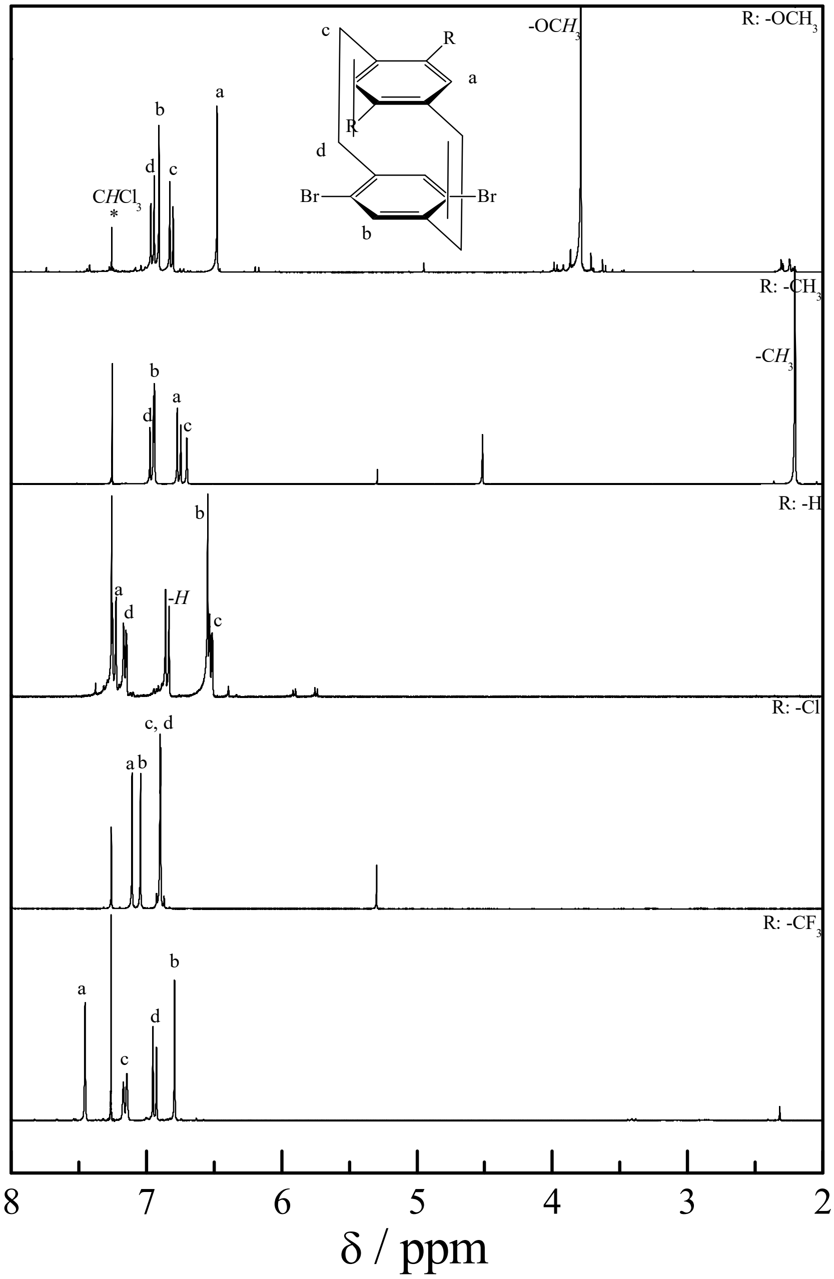

Figure S3. ^{1}H-NMR spectra of compounds 9a-9e (* means the utilized solvent).

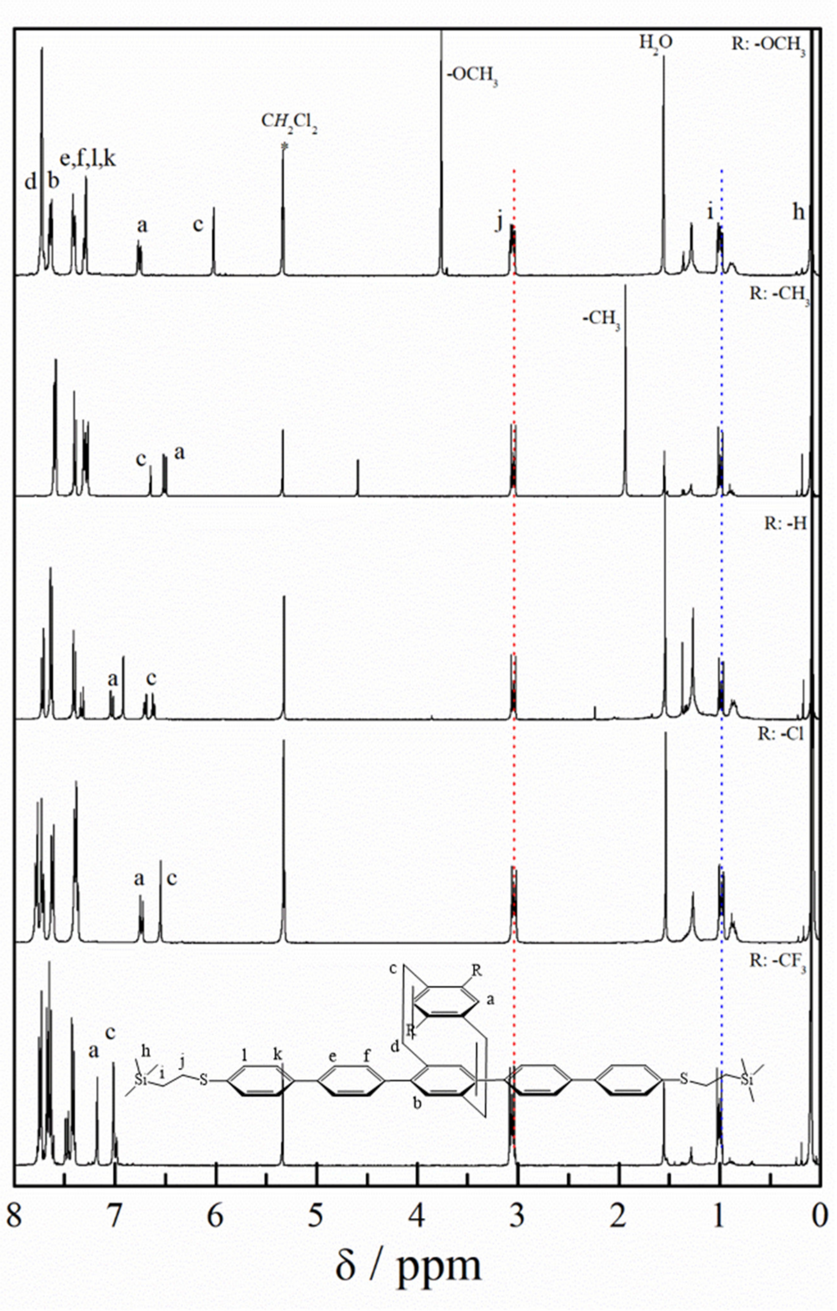

Figure S4. ^{1}H-NMR spectra of compounds 10a-10e (* means the utilized solvent).

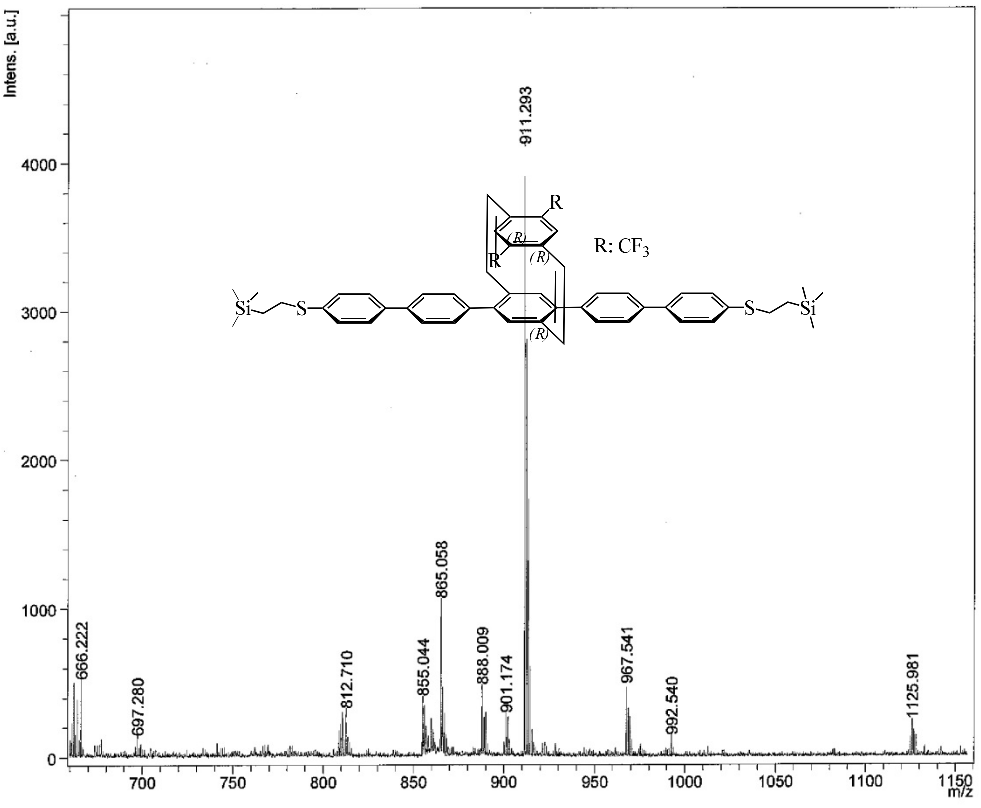

Figure S5. MADITOF-MS spectrum of compound10a.

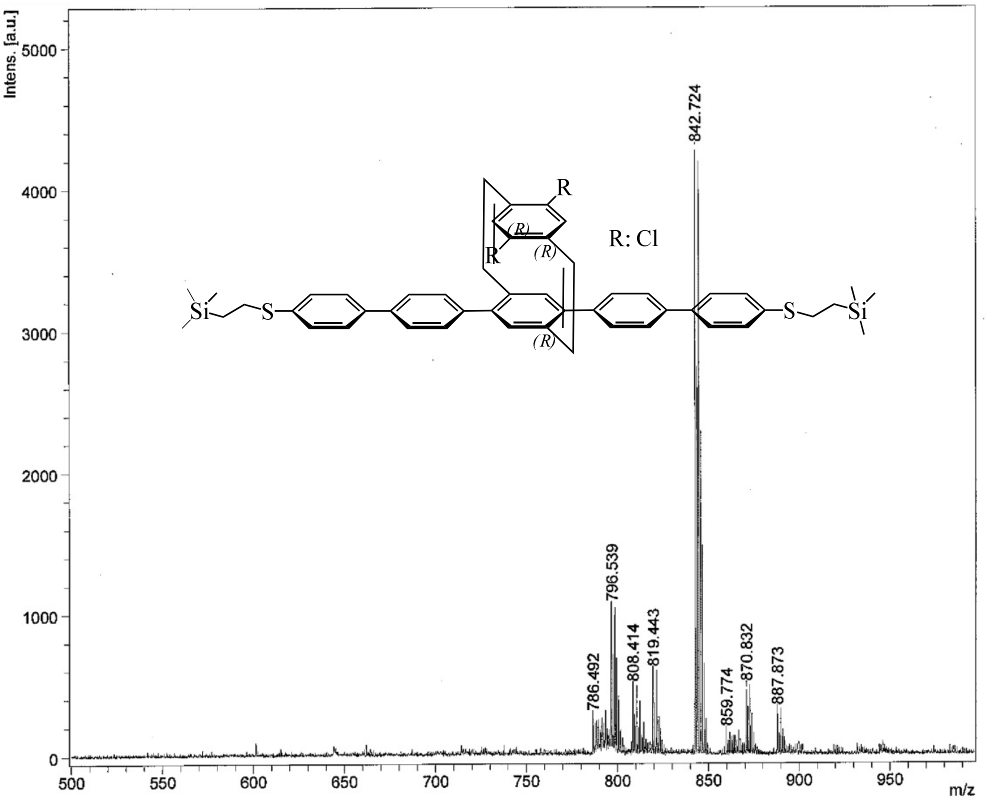

Figure S6. MADITOF-MS spectrum of compound10b.

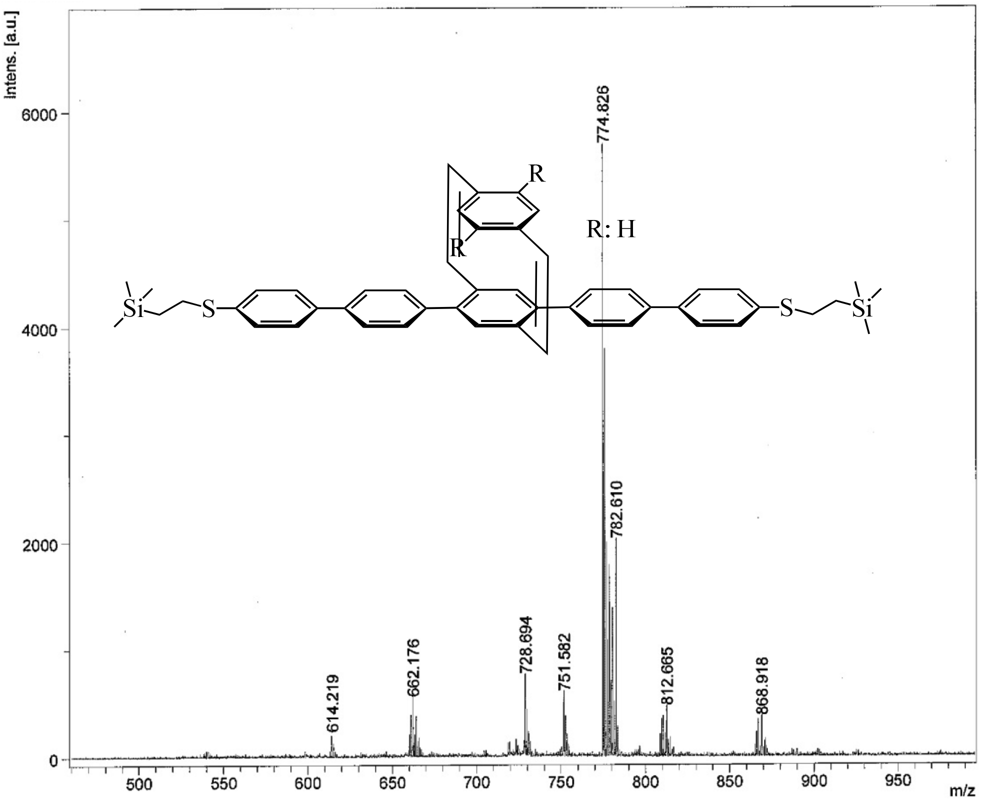

Figure S7. MADITOF-MS spectrum of compound10c.

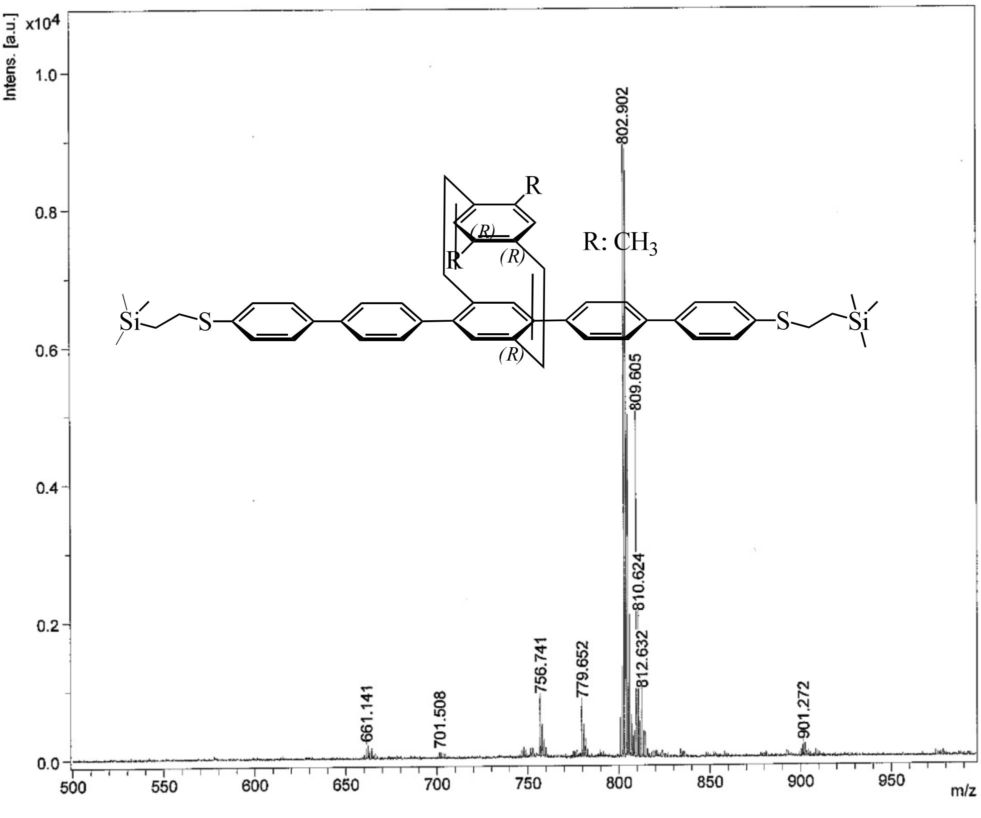

Figure S8. MADITOF-MS spectrum of compound10d.

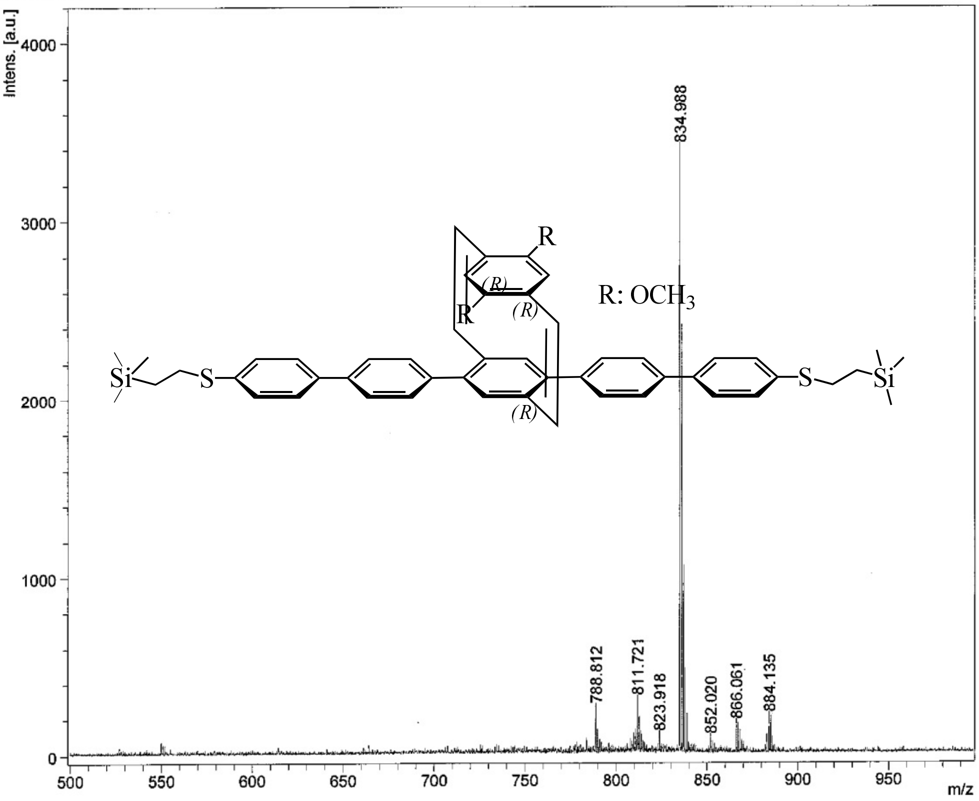

Figure S9. MADITOF-MS spectrum of compound10e.

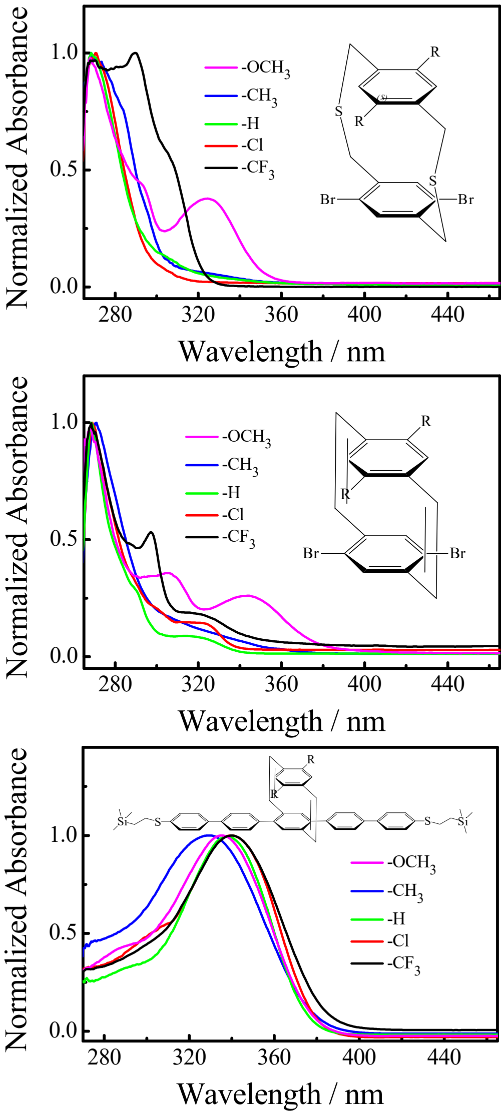

Figure S10. UV-vis spectra of intermediates and the final molecular wires in deuterated chloroform.

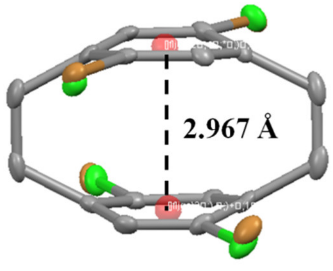

Figure S11. Single crystal structure of paracyclophane-1,9-dienes.

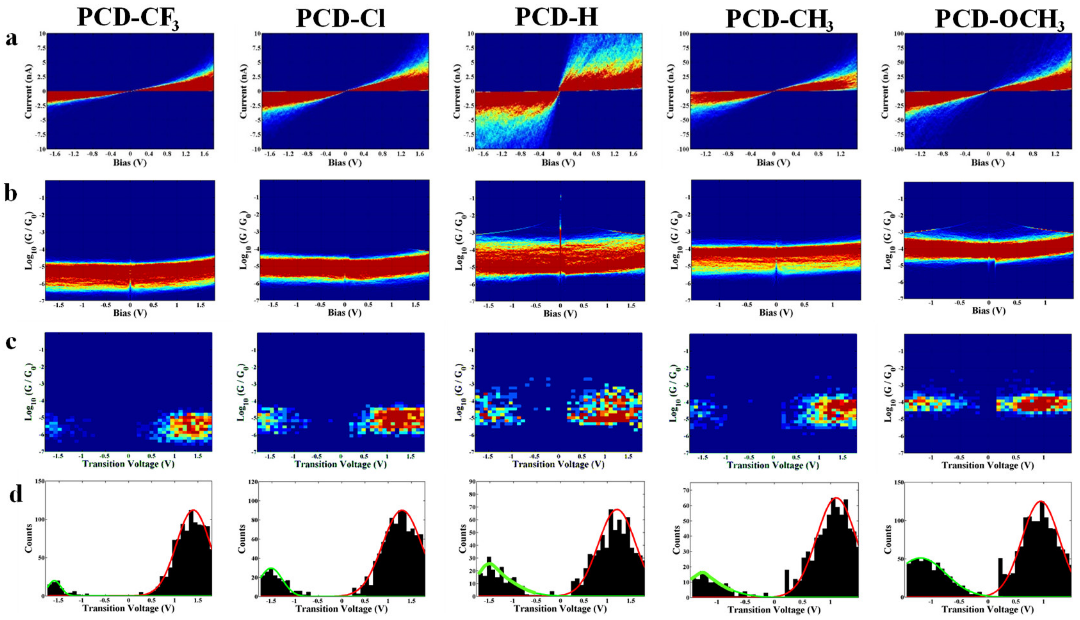

Figure S12. (a) Current-voltage 2D histograms, (b) conductance-voltage 2D histograms, (c) transition voltage 2D histograms, and (d) transition voltage 1D histograms of the five molecular wires.

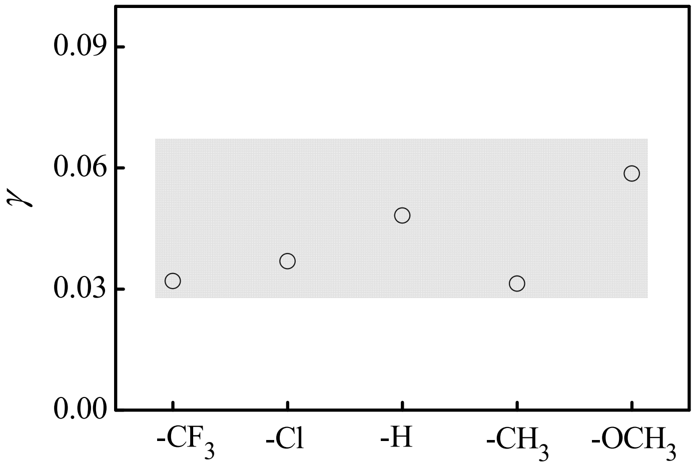

Figure S13. Calculated voltage division factor (γ) for the five PCD molecular wires.

Table S1. Experimental and DFT calculation results for PCD molecular wires and calculated values of φ_0 and γ base on eqs (1) and (2) developed by Baldea.

	$G^{(a)}$ (G_0)	$V_{t,n}^{(a)}$ (V)	$V_{t,p}^{(a)}$ (V)	$V_{t,a}^{(b)}$ (V)	$\varphi_0^{(b)}$ (eV)	$\gamma^{(b)}$	HOMO[c] (eV)	LUMO[c] (eV)
PCD-CF_3	5.8×10^{-6}	1.6	1.38	1.28	1.28	0.032	-5.72	-1.61
PCD-Cl	8.2×10^{-6}	1.53	1.29	1.21	1.21	0.037	-5.59	-1.48
PPC-H	2.5×10^{-5}	1.50	1.20	1.15	1.15	0.048	-5.46	-1.32
PPC-CH_3	3.2×10^{-5}	1.26	1.09	1.01	1.01	0.031	-5.40	-1.32
PPC-OCH_3	8.1×10^{-5}	1.22	0.93	0.91	0.91	0.058	-5.29	-1.20

[a]Data obtained from STM experiments;

[b]Data obtained from calculation according to eqs S1 and S2;

[c]Data obtained from DFT calculation, which is known to sometimes underestimate the gap of the molecule, therefore the bandgap values obtained from that are only good for rough comparisons with the experimental data.
